# Development of the cutaneous microbiome in the preterm infant: A prospective longitudinal study

**DOI:** 10.1371/journal.pone.0176669

**Published:** 2017-04-27

**Authors:** Mohan Pammi, Jacqueline L. O’Brien, Nadim J. Ajami, Matthew C. Wong, James Versalovic, Joseph F. Petrosino

**Affiliations:** 1 Section of Neonatology, Dept. of Pediatrics, Baylor College of Medicine, Houston, United States of America; 2 Alkek Center for Metagenomics and Microbiome Research and Department of Molecular Virology and Microbiology, Baylor College of Medicine, Houston, United States of America; 3 Department of Pathology & Immunology, Baylor College of Medicine, Houston, United States of America; University of Illinois at Urbana-Champaign, UNITED STATES

## Abstract

**Background:**

Neonatal sepsis in preterm infants is often due to organisms that colonize the skin including *Staphylococcus* spp. and *Candida* spp. Development and maturation of the skin microbiome in the neonatal period, especially in preterm infants, may be critical in preventing colonization with pathogens and subsequent progression to neonatal sepsis. Development of the skin microbiome in preterm infants or its determinants in the first 4 weeks of life has not been evaluated.

**Methods:**

We evaluated the skin microbiome from three body sites, antecubital fossa, forehead and gluteal region, in a prospective cohort of 15 preterm (birth weight < 1500 g and < 32 weeks of gestation) and 15 term neonates. The microbiome community membership and relative abundance were evaluated by amplification and sequencing the bacterial V3-V5 region of the16S rRNA gene on the 454 GS FLX platform. We used linear mixed effects models to analyze longitudinal data.

**Results:**

The structure and composition of the skin microbiome did not differ between the three sampling sites for term and preterm infants in the neonatal period. However, skin bacterial richness was positively associated with gestational age in the first four weeks of life. Intravenous antibiotics negatively impacted the bacterial diversity of the skin but we did not see differences with respect to feeding or mode of delivery.

**Conclusions:**

Gestational age, which influences the maturity of skin structure and function, is associated with the development of the preterm cutaneous microbiome. Understanding the maturation of a healthy skin microbiome, prevention of pathogen colonization and its role in the development of immunity will be pivotal in the development of novel interventions to prevent infections in critically ill preterm infants.

## Introduction

The infant skin microbiome undergoes a major transition during the birthing process; the fetal skin *in utero* is bathed in the amniotic fluid, and after birth the skin encounters a gaseous microbe-rich environment. The naïve neonatal cutaneous microbiome matures and evolves rapidly into the adult microbiome during infancy and early childhood [1–3]. At birth, mode of delivery (vaginal or cesarean) influences the microbial composition but the skin microbiota are similar between skin sites [4]. Site-specific maturation of the skin microbiome occurs at about 1–3 months of age while continuing to evolve into a more even and stable community with time [3]. The evolving neonatal skin microbiome may be influenced by the characteristics of the neonatal skin, development of the immune system, and environmental exposures such as antibiotics and unique conditions like the neonatal intensive care (NICU) environment [3,5,6].

The adult skin is colonized by Proteobacteria, Actinobacteria, and Firmicutes in that order of abundance [7,8], whereas the infant’s skin is dominated by Firmicutes followed by Actinobacteria, Proteobacteria, and Bacteroidetes [9]. The skin of the infant and the neonate differs from that of the adult in structure and function [10,11]. The unique characteristics of the preterm skin may influence the development of the microbiome and the absence of maturity and stability of the microbiome may predispose to pathogen colonization and neonatal sepsis [11]. Physico-chemical properties of the skin (pH, temperature, and nutrient availability), barrier integrity (keratinization and intactness), sebaceous secretions and innate immune responses may modify the microbiome. Changes in the skin after birth include reduction of transepidermal water losses, changes in the skin pH and sebaceous activity and increase in water content. In addition, barrier and water handling functions and epidermal cell proliferation and keratinization rapidly mature happen in the first few weeks of life, all of which may affect skin colonization [12]. An intact microbiome with adequate microbial richness and diversity resists colonization by pathogens and a disrupted microbiome may predispose neonates to sepsis. Neonatal sepsis is frequently due to microorganisms colonizing the skin such as coagulase negative *Staphylococcus* spp. (45%), and *Candida* spp. (6%) [13]. The microbiome also plays a significant role in the development of the immune system and dysbiosis may to contribute to immune deficiency or hyperactivity states [14,15]. Although the skin microbiome has been examined in term infants early in the first year [3], it has not been evaluated in preterm neonates in the neonatal period in the NICU.

We sought to characterize the bacterial diversity and community structure of the skin microbiota in preterm infants in the first 4 weeks of life. Our specific aims were to: i) compare the development of the skin microbiome in preterm neonates (< 1500 g and < 32 wks. gestation) to that of term neonates (≥ 37 weeks of gestation) and to ii) investigate potential clinical correlates of the skin microbiome in preterm neonates.

We report on the longitudinal development in the skin microbiome from the antecubital fossa, forehead and gluteal region in this neonatal cohort, focusing on preterm infants in the NICU. Bacterial community richness and diversity were positively associated with gestational age and negatively with antibiotic exposure.

## Methods

### Patient enrollment

The study protocol was approved by the Institutional Review Board at Baylor College of Medicine, Houston, TX, USA. We consented parents after delivery and enrolled 15 preterm and 15 term neonates. Seven of the fifteen term infants were recruited from the newborn nursery and were followed in 2–4 weeks at home by the PI (MP) or at an outpatient clinic at Texas Children’s Hospital (TCH). The other eight term infants and all preterm infants were in an NICU at TCH.

### Skin swabs for assessment of the microbiome and skin colonization

Skin swab specimens were obtained from 3 body sites: the antecubital fossa, forehead and the gluteal region. A 2 by 2 cm area at each of the 3 sites were sampled with a Cytopak, cytosoft brush, CP-5B that had been soaked in sterile 0.15 M NaCl with 0.1% Tween 20 (Fisher Scientific, Fair Lawn, NJ). Term infants were sampled at two time points (in the first 48 hr and between 2–4 weeks of life) and preterm infants at 5 time points (in the first 48 hr and weekly for 4 weeks). Skin swab samples were collected and transported to the laboratory on the day of collection.

### DNA extraction and 16S 454 sequencing data generation

DNA was extracted using the PowerLyzer PowerSoil DNA Isolation kit (MO BIO Laboratories, Carlsbad, CA, USA) with the Human Microbiome Project (HMP) modifications to the manufacturer’s protocol [16]. Samples were processed within two weeks of arriving to the lab. DNA quality and yield were evaluated by agarose gel and Qubit fluorometer (Life Technologies Corporation, Carlsbad, CA, USA). The 16S rRNA gene libraries were generated by the Alkek Center for Metagenomics and Microbiome Research (CMMR) at Baylor College of Medicine, using the V3-V5 (357F/926R) primer in accordance with standard HMP protocols [17]. The 16S rRNA libraries were sequenced by the Human Genome Sequencing Center (HGSC) at Baylor College of Medicine using a Roche 454 GS FLX+ instrument (Roche, Indianapolis, IN) operated with titanium chemistry.

The 16S rRNA gene pipeline data incorporates phylogenetic and alignment-based approaches to maximize data resolution. The resulting reads were truncated to 280 bps and reads with <1 expected error were kept using USEARCH v7.0.1001 [18]. Our pipeline for 16S analysis leverages custom analytic packages developed at the CMMR, as well as several other tools.

16S rRNA gene sequences were assigned into OTUs at a similarity cut-off value of 97% using the UPARSE pipeline in QIIME and the SILVA Database (version 119) [19]. Abundances for non-singleton reads were recovered by tracing the read to its OTU origin via clustering. Singletons were assigned to OTUs via mapping to non-singleton reads at 99% using USEARCH. A custom script constructed an OTU table from the output files generated in the previous two steps, which was then used to calculate alpha-diversity (observed OTUs and Shannon diversity Indices), and provide taxonomic summaries.

Analysis and visualization of microbiome communities were conducted in the statistical platform R [20], utilizing the phyloseq package [21] to import sample data and calculate alpha diversity metrics as well as their means and standard deviations. To assess alpha diversity over time, we applied linear mixed effects modeling in R (R Core Team (2016). R: A language and environment for statistical computing. R Foundation for Statistical Computing, Vienna, Austria. URL https://www.R-project.org/), using the lmer command within the lme4 package to fit models for the observed number of OTUs, and the Shannon diversity index. We included random slopes and intercepts for individual subjects, and evaluated term/preterm status, sampling site and time of sampling in weeks as fixed effects. We also used linear regression to evaluate gestational age (continuous) and alpha diversity outcomes at the time of baseline sampling and calculated Pearson correlations. We performed hierarchical clustering for samples based on relative abundance of taxa, visualized with the ‘pheatmap’ package in R. We used principle coordinate analysis (PCoA), based on the weighted and unweighted UniFrac distance to visualize between-sample differences (beta-diversity), and tested for between-site differences with the PERMANOVA test. Our threshold for significance was p < 0.05.

## Results

### Characteristics of the enrolled cohort

We enrolled 15 preterm neonates (mean ± SD (range): birth weight: 1015 ± 269 g (665–1417 g), gestational age: 27 ± 2.6 weeks (range 24 1/7 to 32 1/7 wk) (Table 1). All preterm neonates were in the NICU for the duration of the study. We also enrolled a comparative group of 15 term neonates (mean ± SD (range): birth weight: 3188 ± 538 g (1885–3785 g), gestational age: 38.6 ± 1.3 wk (range 37 1/7 to 40 5/7 wk). Of these term neonates, 7 were enrolled from the newborn nursery and went home in the first few days of life and had no medical problems. The first set of swabs for these infants were collected in the newborn nursery and the second set was collected at home by the principal investigator MP, except for one neonate who visited our follow-up clinic. Eight term neonates had medical problems including gastroschisis and congenital heart disease that necessitated their stay in the NICU, and swabs were performed at admission and between 2–4 weeks in the NICU.

**Table 1:** Characteristics of enrolled patients.

	Preterm (n = 15)	Term (n = 15)
**Gestational age in wks** **Mean (SD)**	27.29 (2.66)	38.52 (1.11)
**Birth weight in grams** **Mean (SD)**	1010.40 (277.95)	3105.93 (569.76)
**Sex**	M:F = 9:6	M:F = 9:6
**Location**	All NICU	NICU 8, Nursery 7
**Mode of delivery**	CS: Vag = 10:5	CS: Vag = 7:8
**Chorioamnionitis**	4	none
**Antibiotics in the first 48 hrs of life**	12	5 of 15
**Antibiotic duration in days** **Mean (SD)**	6.60 (6.25)	0.87 (1.25)
**Nutrition**	BM 10, 1 formula, PN-5	BM 12, 2 formula, PN-1
**Late-onset sepsis**	6	none
**NEC Stage II or III**	4	none

NICU- neonatal intensive care unit, Del: mode of delivery, CS- cesarean section, Vag- vaginal delivery, PN- parenteral nutrition, BM- breast milk, NEC- necrotizing enterocolitis.

### Assessment of the neonatal skin microbiome

Microbial DNA from a total of 309 skin swabs from 30 subjects was extracted and sequenced. After quality trimming and filtering, 280 samples remained, which yielded 1,221,507 reads. Samples were subsequently rarefied to 1,000 reads that were selected based on a bacterial richness rarefaction curves, which resulted in the exclusion of 41 additional samples. For this study, a total 235 samples from 29 subjects (15 preterm and 14 term neonates) were analyzed.

#### Longitudinal and site-specific development of the skin microbiome

**Fig 1:**
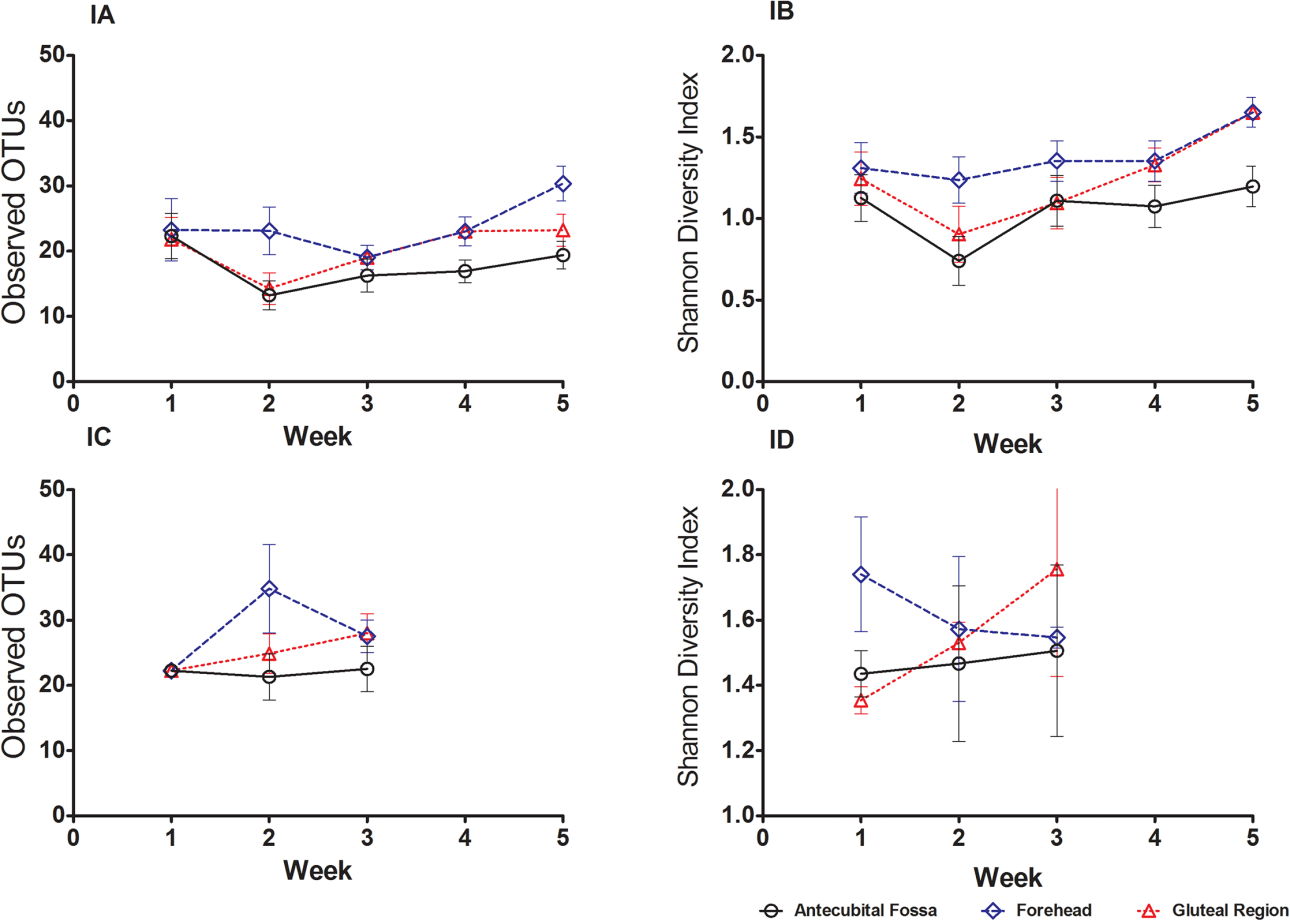
Longitudinal and site-specific development of the cutaneous microbiome in preterm and term infants. Observed Operational Taxonomic Units (OTUs) (mean ± SEM) and Shannon diversity index (mean ± SEM) for preterm (Fig 1A and 1B) and term infants (Fig 1C and 1D) are plotted by sampling week and skin site. Term infants were sampled at 2 time points (birth and between 2–3 weeks of age) and preterm infants every week from birth for 5 time-points. For each skin site, the number of OTUs and Shannon diversity index in preterm infants decreased between week 1 and week 2, and then increased over time until week 5. The trend for diversity over time was not significantly different between the three sites in our linear mixed effects model for observed OTUs and Shannon diversity index in preterm infants. Overall, bacterial diversity tended to be higher for term infants compared to preterm infants (p = 0.04 for main effect of term vs. preterm status for Shannon diversity index; p = 0.05 for observed number of OTUs).

We considered the trajectories of preterm and term infants separately, given their different sampling schedules. For preterm infants, measurements of bacterial richness in the skin showed a decreasing tendency between the first and second week of sampling (Fig 1A and 1B), particularly in the antecubital fossa and gluteal region, followed by an increase in the weeks thereafter. However, we tested for a linear trend over time in the preterm infants using linear mixed effects models, and found no statistically significant differences between the three sites, for either bacterial community richness (number of observed Operational Taxonomic Units [OTUs] counts, Fig 1A), or community richness and evenness (Shannon diversity index, Fig 1B) (both p > 0.05 in linear mixed effects model). The term infants were followed over two timepoints, and there was no change in either alpha diversity metric over time although limited sample size and uneven sampling precluded significance testing with linear mixed effects models (Fig 1C and 1D). Overall, bacterial diversity tended to be higher for term infants (p = 0.04 for main effect of term v. preterm status for Shannon diversity index; p = 0.05 for observed number of OTUs).

We found no significant differences between the three sites in terms of beta-diversity (i.e., between-sample diversity), as assessed by the weighted unweighted UniFrac metrics, at any timepoint. Figures A1 and A2 in S1 File show PCoA plots at each timepoint for both term and preterm infants for the weighted UniFrac metric. Results were similar with unweighted UniFrac (results not shown). We evaluated the rate of change of the structure and composition of the skin microbiome (Figure B in S1 File) by calculating the unweighted and weighted UniFrac distances between each sample and its baseline sample (week 1 sample performed in the first 48 hrs. of life). For preterm infants, the mean weighted and unweighted UniFrac distances remained constant between each week and the baseline sample.

**Fig 2:**
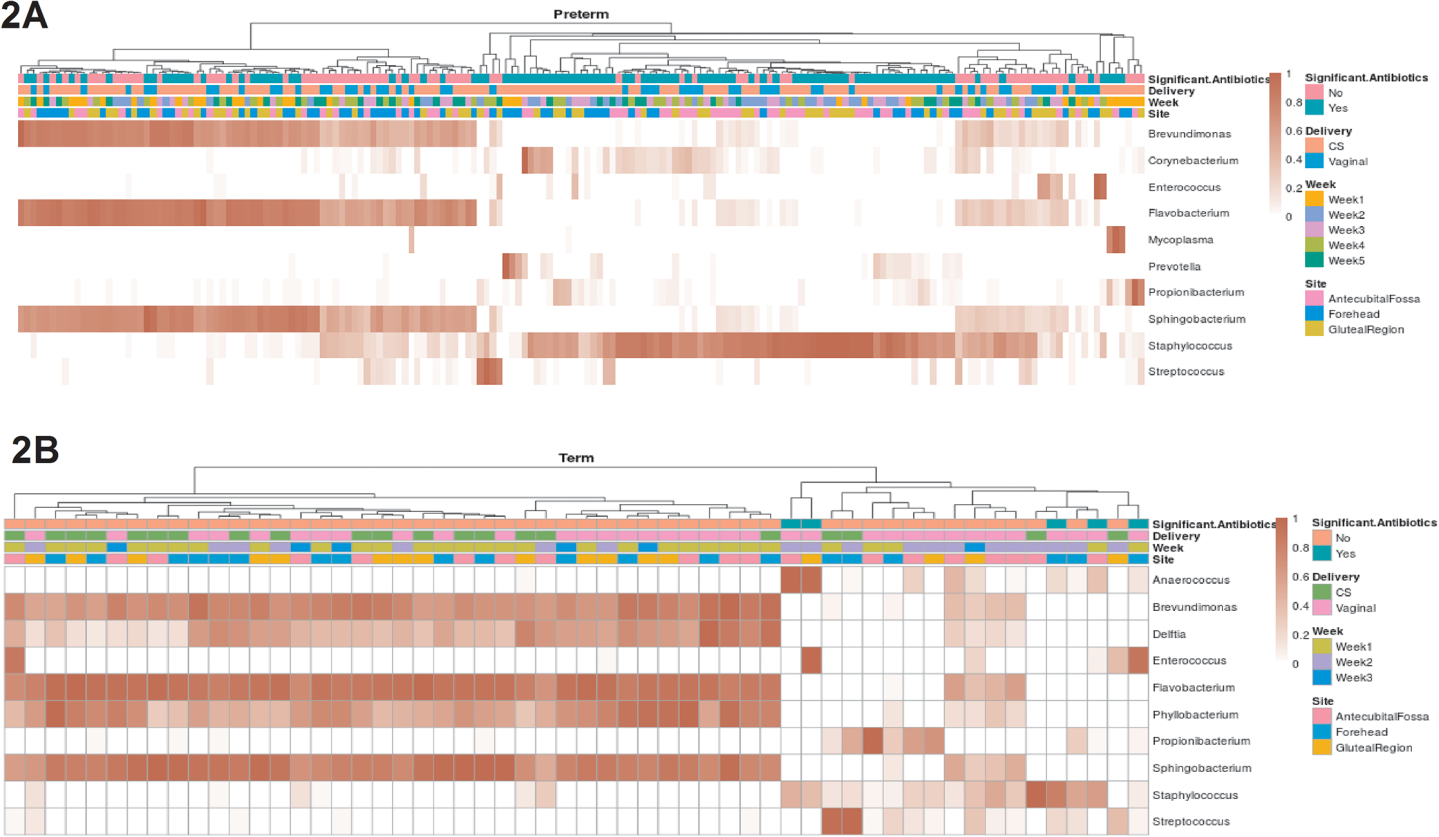
Hierarchical clustering for preterm and term infants, based on relative abundance at the genus level. Each column represents a sample, with darker red indicating higher relative abundance. The top 10 most abundant genera are shown for preterm (2A) and term (2B) infants, separately. The top four rows show metadata for each sample namely use of significant antibiotics, mode of delivery, week of sample collection and sampling site.

We used hierarchical clustering to examine the relative abundance of genera in preterm and term infants separately (Fig 2A and 2B). When we considered all samples together, Firmicutes was the dominant phylum (mean relative abundance = 40%), followed by Bacteroidetes (mean relative abundance = 39%), Proteobacteria (mean relative abundance = 11%), and Actinobacteria (mean relative abundance = 7%). Preterm skin microbiome had an increased relative abundance of Firmicutes relative to that of Bacteroidetes compared to term neonates. The predominant genera in the skin microbiota were *Staphylococcus* (phylum Firmicutes) followed by *Flavobacterium* (phylum Bacteroidetes), *Sphingobacterium* (phylum Bacteroidetes) and *Brevundimonas* (phylum Proteobacteria). The limited sample size and varied timing of sample collection precluded significance testing between groups defined by term/preterm status and sampling site. An increased mean relative abundance of *Staphylococcus*, *Corynebacterium* and *Prevotella* relative to *Brevundimonas*, *Flavobacterium* and *Sphingobacterium* was found in preterm neonates relative to term neonates.

#### Gestational age and the diversity of the cutaneous microbiome

**Fig 3:**
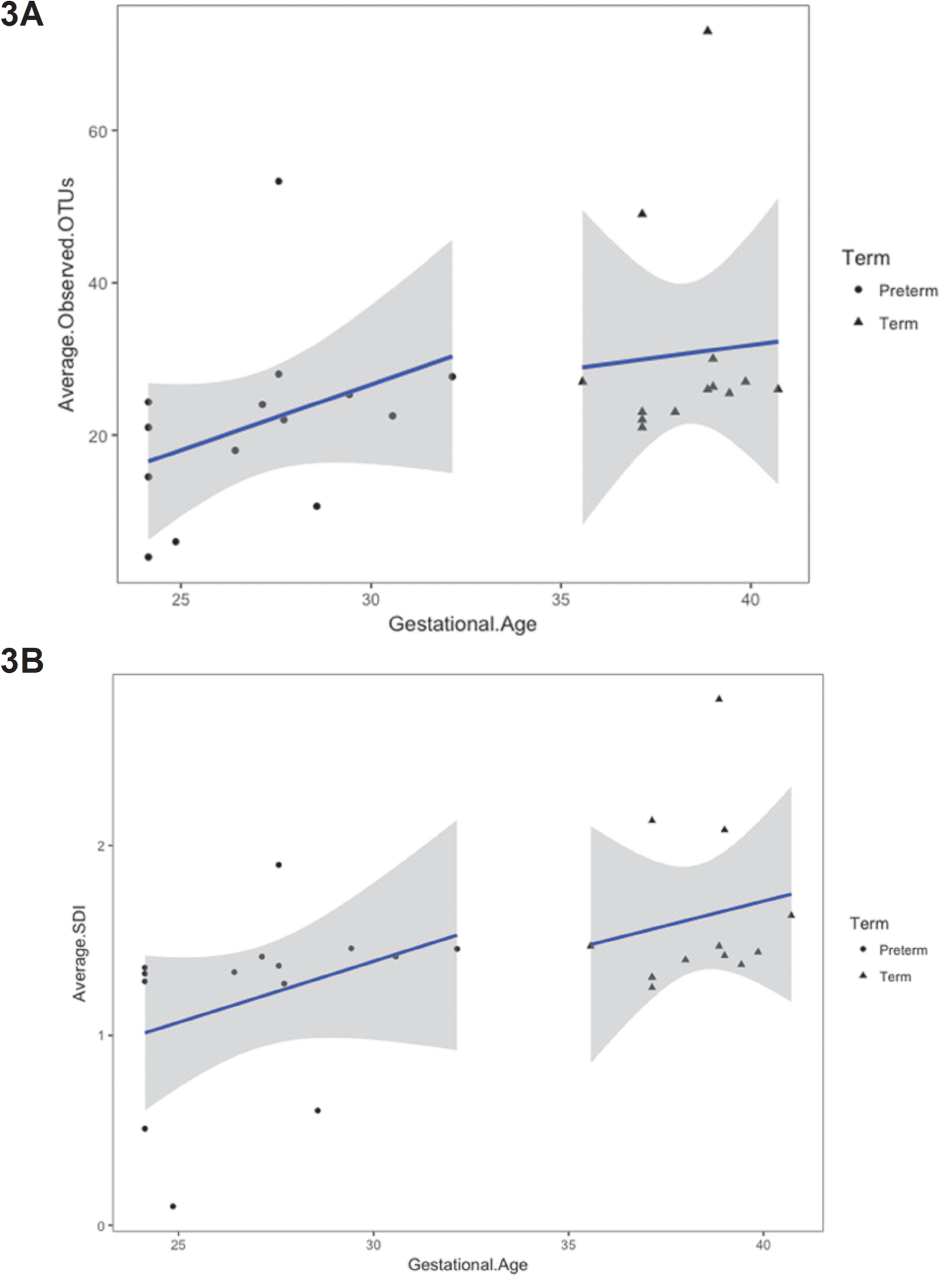
Gestational age is associated with the skin microbiome (at baseline) in preterm infants. **3A. Average Observed OTU:** Each dot represents the average number of Operational Taxonomic Units (OTUs) for the subject, across the sites. The average OTU is linearly regressed against gestational age at baseline samples and shaded areas are the 95% confidence intervals for the regression line. Gestational age is significantly associated with average number of OTUs, when both preterm and term infants are considered together (R = 0.39, p = 0.04). **3B. Average Shannon diversity index**: Each dot represents the average Shannon diversity index for the subject, across the sites. The average Shannon diversity index is linearly regressed against gestational age at baseline samples and shaded areas are the 95% confidence intervals for the regression line. Gestational age is significantly associated with average Shannon diversity index, when both preterm and term infants are considered together (R = 0.49, p < 0.009).

Gestational age was positively associated with bacterial richness. Considering that we did not observe a significant difference in community richness between sampling sites, we calculated the average observed OTUs for each subject at baseline (0–48 hr of life). At baseline, there was a positive association between gestational age of the infant and the average number of observed OTUs (Fig 3A, p = 0.04), as well as a positive association between gestational age and the average Shannon diversity index (Fig 3B, p = 0.009) at baseline. In addition, considering all time points, there was also a significant positive correlation with corrected gestational age with OTUs and Shannon diversity index for the three sampled sites using linear mixed effects model (p < 0.05, Figures D1 and D2 in S1 File).

#### Clinical factors that may influence the microbiome

**Fig 4:**
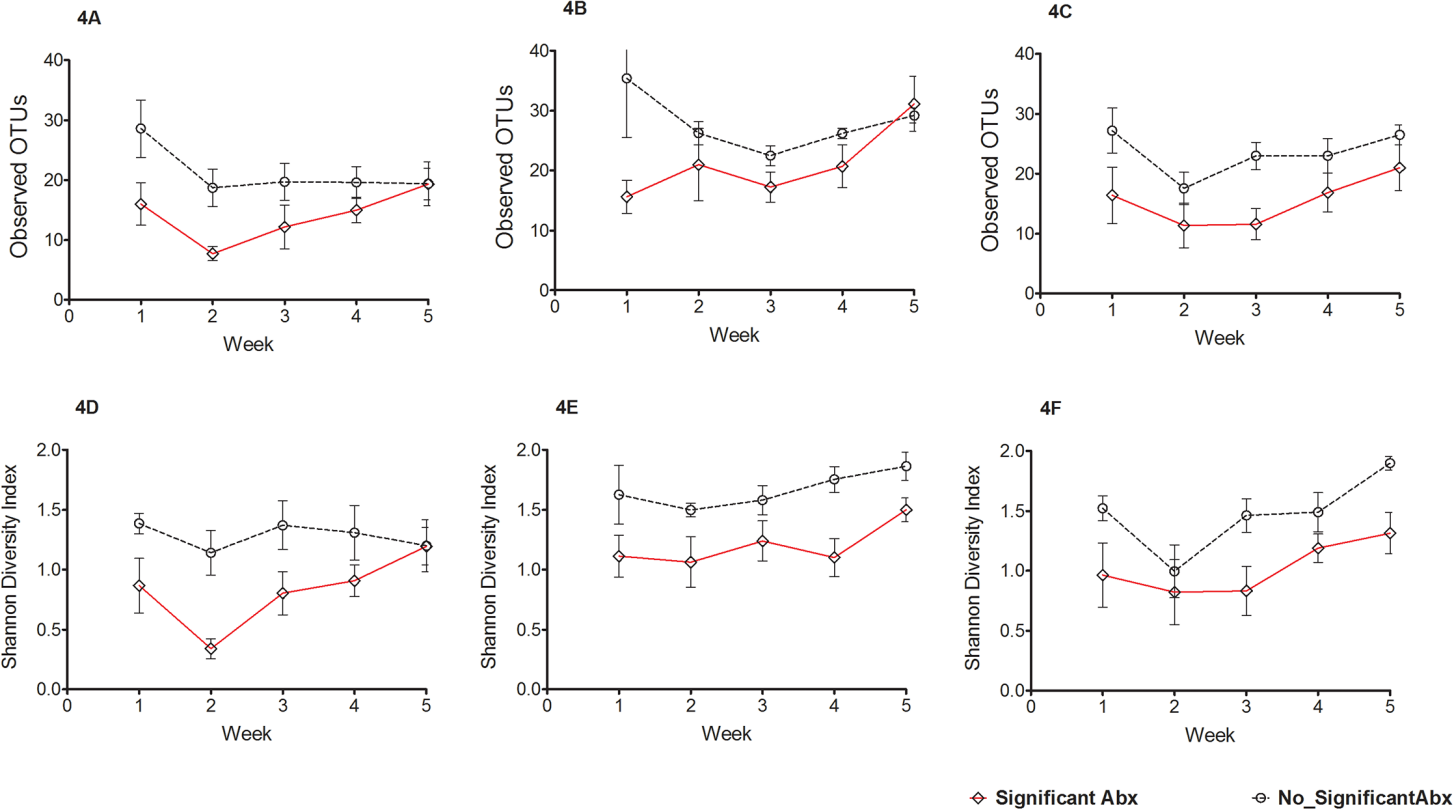
The effects of antibiotic exposure on the development of the skin microbiome. Alpha diversity indices of operational taxonomic units (OTUs) (mean ±SEM) are depicted in panels A, B and C for antecubital fossa, forehead and gluteal region respectively and Shannon diversity index (mean ±SEM) in panels D, E and F for antecubital fossa, forehead and gluteal region respectively in preterm neonates who were exposed to significant antibiotics (> 48 hr) compared to those not exposed. Exposure to significant antibiotics decreases alpha diversity measures in the preterm skin microbiome in the neonatal period.

We evaluated the effects of antibiotics on the developing cutaneous microbiome and found significantly lower community richness over the sampling period in certain sites in infants who had received significant antibiotics compared to those who did not (significant antibiotics defined as > 48 hr of antibiotics (Fig 4). We analyzed longitudinal trends of OTU richness using linear mixed effects models for each site and found significant interaction between significant antibiotics and time for antecubital fossa (p = 0.02) but not for forehead (p = 0.08) or the gluteal region (p = 0.34). The effects of the duration of antibiotic use on the developing skin microbiome by categorizing duration of antibiotics as low (3–10 days), medium (11–14 days) and high (> 14 days) (Figure E in S1 File). Infants in the ‘low duration of antibiotics’ category tended to have higher community richness and evenness, although results did not reach statistical significance. We investigated the effects of feeding type (i.e., maternal breast milk, donor milk feeding, formula feeding or predominantly total parenteral nutrition) on the richness and evenness of the skin microbiome and did not find any statistically significant differences in alpha diversity over time between these four groups (p>0.05) (Figure F in S1 File). We did not observe differences by mode of delivery in microbial richness, diversity, or taxonomic profiles in the five weeks after birth of the cutaneous microbiome (Figure G in S1 File).

## Discussion

We report our prospective, longitudinal study of the preterm skin microbiome in the neonatal period in comparison to term infants. We did not see differences in community richness or taxonomic composition between the sampling sites (antecubital fossa, forehead and gluteal region). Preterm neonates exhibited decreased diversity and community evenness compared to the term infant. There were time-trends of alpha diversity increasing with gestational age. The most abundant phyla in the skin of preterm neonates were Firmicutes and Proteobacteria and the predominant genus was *Staphylococcus*. We did not find significant correlation with type of milk feeding or mode of delivery but our study was not powered to detect these associations. To our knowledge this is the first study to describe the longitudinal development of the cutaneous microbiome in preterm neonates.

We did not observe site-specific differences between antecubital fossa, forehead or the gluteal region in alpha diversity metrics or microbial profiles. In adults, the three skin sites we chose differ in topographical and environmental characteristics, which foster distinct microbial communities [2]. The skin of the face and the forehead are rich in sebaceous glands that allow the growth of lipophilic organisms e.g. *Propionibacterium species* (Actinobacteria). The gluteal region with its moist and warm environment fosters Gram-negative bacilli (Proteobacteria) and *Staphylococcus aureus* (Firmicutes) and the antecubital fossa of the forearm, a dry desiccated area, with fluctuations in temperature, harbors diverse organisms but with decreased abundance of all organisms in adults. Similar site-specific differences were reported in a study of the skin microbiome in the first year of life in healthy infants [3]. In the same study, *Propionibacterium* was among the top 5 genera isolated from the forehead (although sebum production was thought to be low) and species belonging to *Finegoldia*, *Clostridium* and *Bacteroides* were isolated from the gluteal region due to its proximity to the gastrointestinal tract. However, we did not find any such site-specific differences in in community richness or microbial diversity but only minor differences in microbial profiles in the preterm neonate. Immaturity of the skin structures and its accessories such as sweat and sebaceous glands and a unique NICU environment may explain the absence of site-specific differences in the neonatal skin microbiome.

Alpha diversity metrics were linearly related to gestational age at baseline and to corrected gestational age during the first 4 weeks of life. Gestational age dependent changes in the intestinal microbiome have been recently reported [22]. The observed association may be explained by the host factors related to the preterm neonate or to the characteristics of the microbiologically confined NICU environment. In the preterm neonate, the immaturity of the skin (increased water content, changes in pH) or its functions (poor barrier function, decreased innate immunity) may have contributed at least in part to gestational age-dependent differences. Moreover, the stratum corneum is thin in preterm neonates and takes 2–3 weeks to mature to that of a term infant [23]. The co-morbidities in the preterm infants, all related to the gestational age, including respiratory illness, sepsis, NEC, use of antibiotics and invasive clinical procedures (e.g. catheter placement) may also have impacted the skin microbiome. All the preterm infants were in the NICU, which is a microbiologically confined environment with rigorous infection control practices including strictly enforced hand hygiene measures and cohorting of pathogen colonized infants. Handling by healthcare staff, exposure to equipment such as incubators and to the microbes associated with the NICU environment may also have modified the development of cutaneous microbiota in preterm neonates [5].

The longitudinal development of the skin microbiome has not been reported before. Although we did not find a significant linear trend, the number of OTUs and Shannon diversity index score tended to decreases from week 1 to week 2, and increase during the subsequent weeks, at least in the preterm infants These temporal trends may indicate the dynamism of the skin microbiome observed early in life analogous to what is observed in the gut microbiome [24]. The structure and composition of the skin microbiome remains constant or undergoes the same rate of change across all weeks as observed by consistent beta-diversity metrics. All preterm infants were nursed in the NICU, whose unique environmental influence may have played a role in the consistency of the microbial profiles seen during the neonatal period.

Taxonomic profiling showed that Firmicutes was the dominant phylum and *Staphylococcus* (phylum Firmicutes) and *Flavobacterium* (phylum Bacteroidetes) were the dominant genera. Preterm skin microbiome had increased abundance of the Firmicutes and decreased abundance of Proteobacteria compared to term neonates. An increased abundance in species of *Staphylococcus*, *Corynebacterium* and *Prevotella* and decreased abundance in species of *Brevundimonas*, *Flavobacterium* and *Sphingobacterium* were found at the genus level in preterm neonates. Skin colonization patterns may influence inflammatory skin conditions and the development of skin immunity. Immune responses to colonizing *Staphylococcus* species may be responsible for erythema toxicum of the newborn, a common benign condition seen in term newborn [25]. Commensal bacteria and their products including staphylococcal lipoteichoic acid or other epitopes may induce skin inflammatory responses and enhance immunity [26]. Secretion of proteases by commensal bacteria, *S*. *epidermidis* prevents colonization with pathogenic *S*. *aureus* [27]. Proteases and other bacterial products may also influence the skin barrier function [23,28]. Predominance of Firmicutes and *Staphylococcus* in the immediate post-natal skin microbiome may be a developmental phenomenon that promotes the development of the skin immune function.

We investigated important clinical correlates that may impact the development of the cutaneous microbiome in preterm infants. The effects of antibiotic therapy on the developing cutaneous microbiome were investigated. The antibiotic protocol in our NICU recommends ampicillin or vancomycin along with gentamicin for empirical therapy of sepsis. We observed a significant impact of antibiotic therapy for more than 48 hr (significant antibiotic exposure) on the developing cutaneous microbiome in preterm neonates. The first skin sampling for microbiome assessment was performed in the first 48 hr and mostly after the first dose of antibiotic therapy and hence the pre-antibiotic microbiome assessment could not be performed. Significant antibiotic exposure decreased community richness and diversity in the 3 sampled skin sites. We also observed decreasing trends in OTUs and Shannon diversity index with increasing duration of antibiotics. Microbial profiles showed decreased abundances of Firmicutes and increased abundances of Bacteroidetes and Proteobacteria after antibiotics. Microbial profiling at the genus level showed increased abundance of *Staphylococcus*, *Propionibacterium* and *Prevotella* and reduced abundances of *Brevundimonas*, *Flavobacterium*, *Sphingobacterium and Corynebacterium*.

We did not find any differences in alpha diversity or microbial profiles with type of feeding namely maternal breast milk, donor milk feeding, formula feeding or predominantly total parenteral nutrition on the development of the skin microbiome. Human milk constituents including milk oligosaccharides promote the growth of Bifidobacteria and Lactobacilli, which modify the intestinal microbiome but its effects on the cutaneous microbiome are not known and we did not find differences [29,30]. We did not observe differences in the cutaneous microbiome by mode of delivery in microbial richness, diversity or taxonomic profiles in the four weeks after birth of the cutaneous microbiome even when neonates were subgrouped by gestational age or by chronological age in weeks. Our findings are similar to other studies that do not show changes in microbial composition of the gastrointestinal microbiome in early life or the placental microbiome based on mode of delivery [22,31]. Aagard *et al* demonstrated that the unique placental microbiome was not influenced by vaginal Group B *Streptococcus* colonization or mode of delivery but by remote antenatal infections [31]. Tarr and co-investigators reported that gastrointestinal microbiota in early life followed a predestined microbial composition irrespective of mode of delivery but whose progression was determined by gestational age [22]. Dominguez-Bello evaluated the oral, nasopharyngeal and skin microbiome immediately after birth and in the meconium in the first 24 hr of life, a single time-point evaluation after birth [4]. Our study was a longitudinal study of the skin microbiome during the first 4 weeks of life and the differences found by mode of delivery at birth may have been diluted by environmental influences and skin characteristics in the preterm infant. Similar to our study, the skin microbiome did not differ by mode of delivery in infants aged 1–3 months of life indicating the dynamic nature of the developing skin microbiome in the first months of life [3]. Our neonatal unit strongly encourages kangaroo care (skin to skin contact) by parents in all preterm neonates and hence we were unable to investigate microbiota differences related to kangaroo care in preterm neonates. Bathing with cetaphil (non-antimicrobial) soaps twice weekly did not influence community richness, diversity indices or microbial profiles during the neonatal period. None of the enrolled neonates were exposed to bathing with antimicrobial soaps including chlorhexidine.

The strengths of our study are the longitudinal sample collection, robust sequencing methodology and investigation of important clinical correlates that potentially can impact the development of the cutaneous microbiome. Limitations include the observational nature of this study and the many confounding clinical variables that may influence the developing skin microbiome. We did not collect environmental sample controls or from caregivers for controls which could have given us clues regarding the origins of the skin microbiota. We were under powered to detect the impact of clinical variables (e.g. NEC, sepsis, feeding and mode of delivery) on the developing cutaneous microbiome.

We report the results of our prospective, cohort study on the longitudinal development of cutaneous microbiota of the skin across multiple sites in the neonatal period. Gestational age was positively associated with microbial diversity of the skin. Other significant predictors of the development of the skin microbiome included antibiotic exposure and its duration. The development of the skin microbiome may critically affect the development of the skin immunity and the maturation of the skin structures and function. A randomized trial to restore skin microbiota by inoculating the skin of infants born after cesarean section with the mother’s vaginal microbiota has been registered (ClinicalTrials.gov Identifier: NCT02407184, www.clinicaltrials.gov). Pilot data from this trial indicating partial restoration of microbiota by this novel transfer method has been recently reported [32]. We strongly believe that the results of our study will spur the development of interventions that aim to optimize the neonatal cutaneous microbiome (skin targeted prebiotic or probiotic therapies) [33], which may prevent pathogen colonization and subsequent systemic infection in an extremely vulnerable population.

## Supporting information

S1 File File has supplementary figures and tables. (DOCX)
