# Supplementary material for: Development of the cutaneous microbiome in the preterm infant: A prospective longitudinal study
Source: PLoS One. 2017 Apr 27;12(4):e0176669. doi: 10.1371/journal.pone.0176669 (PMC5407830; doi:10.1371/journal.pone.0176669)
Supplement: S1 File — (DOCX) [file pone.0176669.s001.docx]

**S1 File**

**Figure A: Beta-diversity as assessed by Weighted UniFrac does not differ by sampling site in preterm or term infants in the first weeks of life**

**Figure A1. Preterm (Weighted UniFrac)**

**
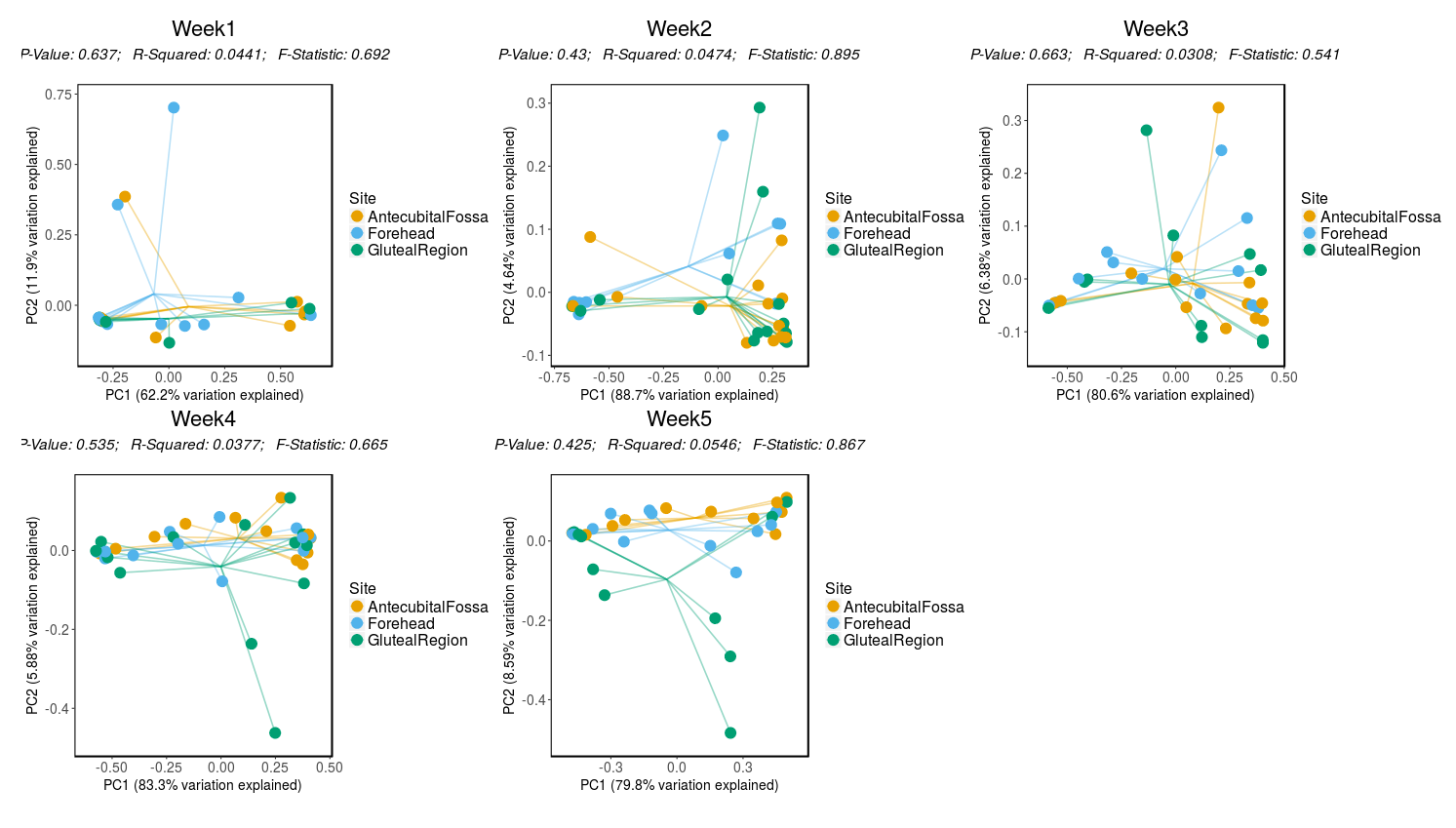
**

**Figure A2. Term (Weighted UniFrac)**


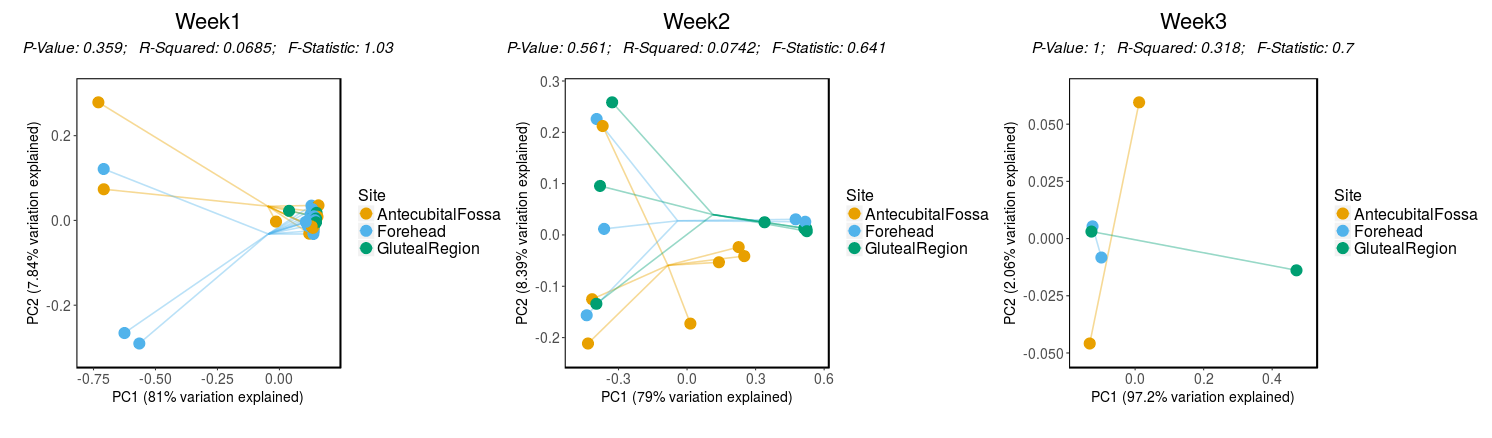


**Figure A legend**: PCoA plots using the weighted UniFrac metric, for preterm (Fig A1) and term (Fig A2) infants at individual timepoints. There were no significant differences between the three sites (antecubital fossa, forehead, gluteal region), in terms of beta-diversity (i.e., community structure and composition). We saw similar results using the unweighted UniFrac metric for preterm and term infants.

**Figure B: β-diversity by UniFrac distances of preterm skin microbiome remain constant in the first weeks of life**

**
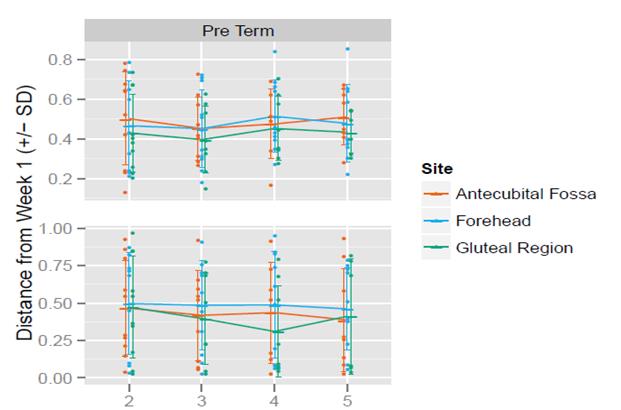
**

**Figure B legend:** Unweighted (top panel) and weighted (bottom panel) UniFrac distances (mean ± SD) of between each sample and its baseline sample (week 1 sample performed in the first 48 hrs. of life) in preterm neonates. Each dot represents the distance between an individual’s sample and their week 1 sample. For preterm infants, the mean weighted and unweighted UniFrac distances remained constant between each week and the baseline sample.

**Figure C: Microbial profiles in preterm and term infants in the first weeks of life**

**Fig C1 – Top 14 Most abundant genera in Preterm infants by site**

**
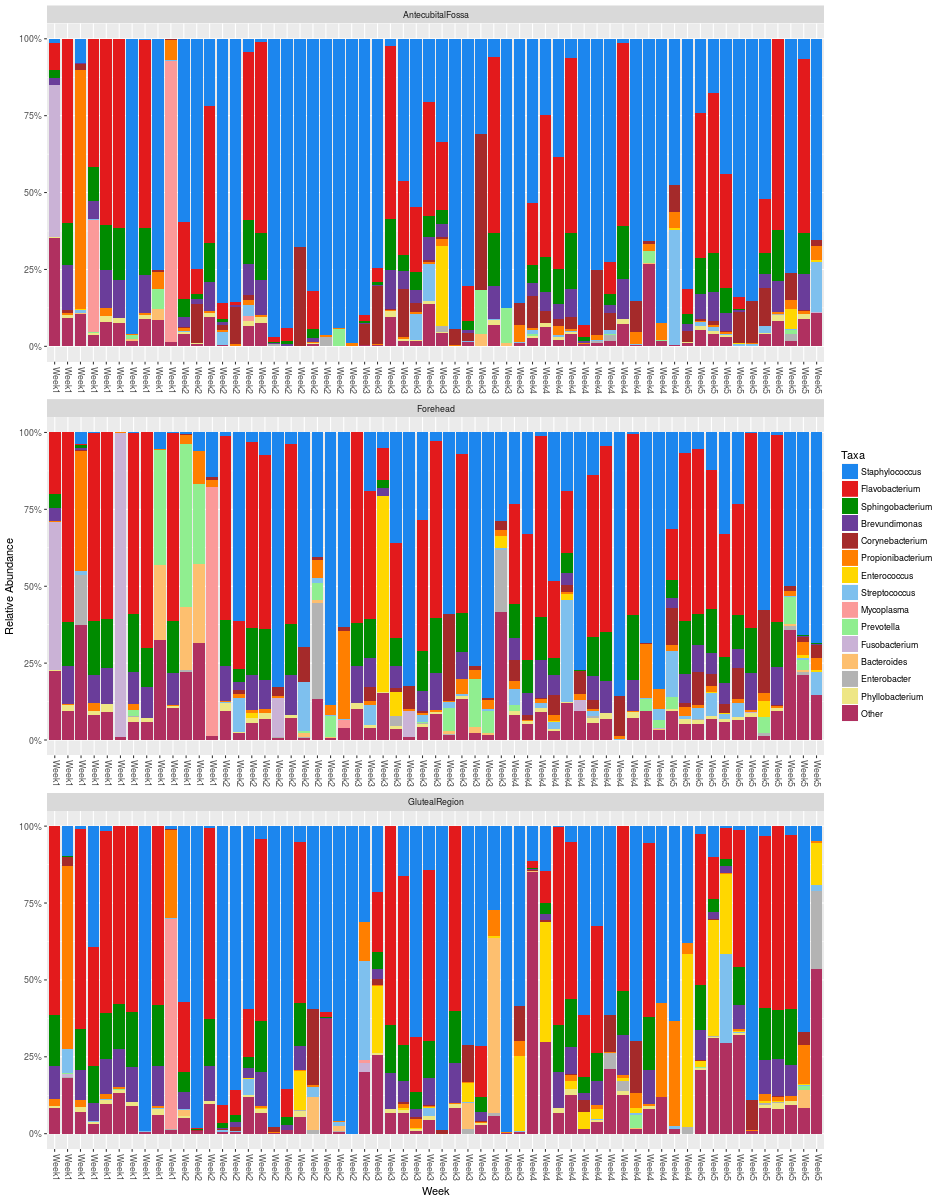
**

**Fig C2- Top 14 Most abundant genera in Term infants by site**

**
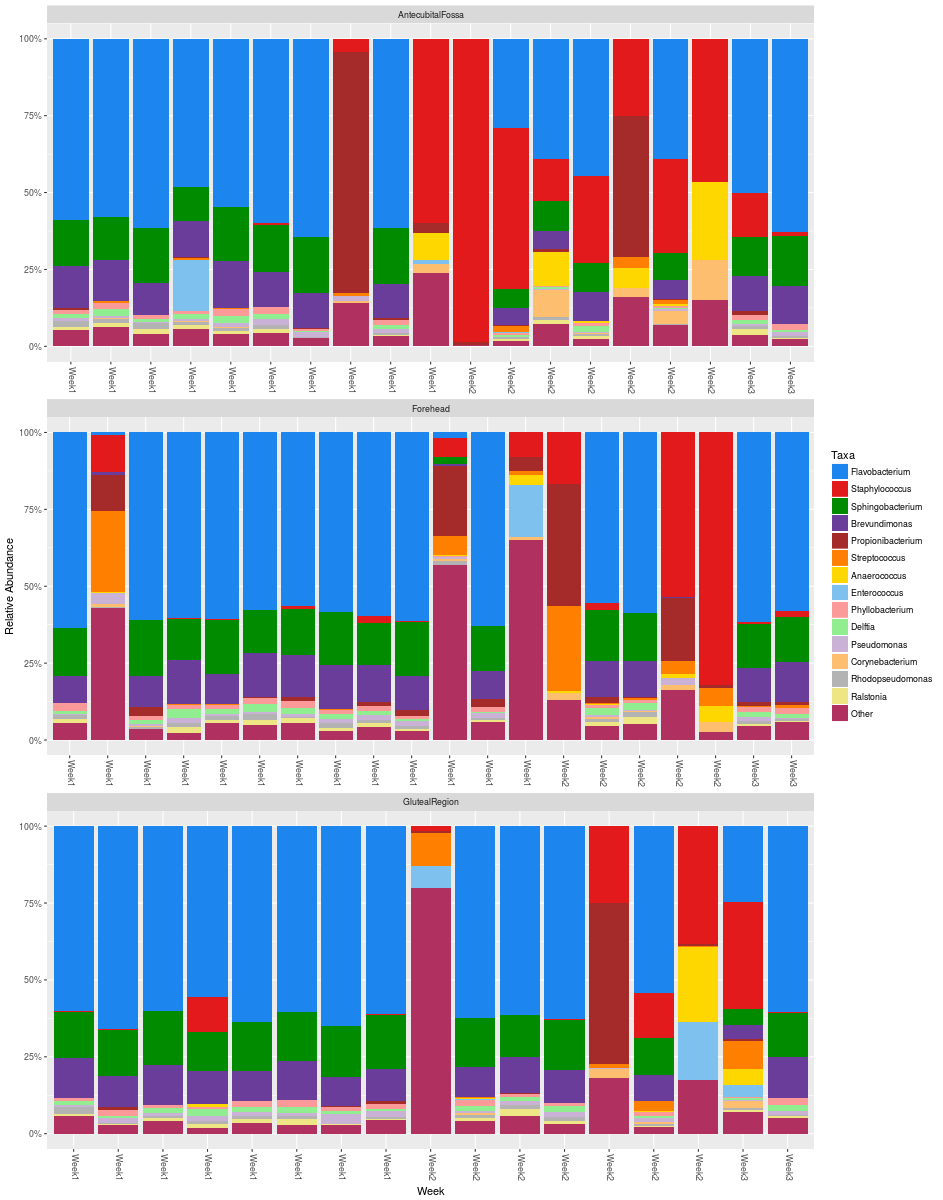
**

**Figure C legend**

**Fig C1: Microbial profiles associated with the skin microbiome in the first 4 weeks of life in preterm infants.** Relative mean abundance of the top 14 most abundant genera is plotted over time, for preterm infants is depicted over age in weeks in the 3 panels, antecubital fossa, forehead and gluteal region respectively from top to bottom. Taxonomic profiling showed that *Staphylococcus* was the dominant genus of the microbiota followed by *Flavobacterium.*

**Fig C2:** **Microbial profiles associated with the skin microbiome in the first 4 weeks of life in term infants.** Relative mean abundance of the top 14 most abundant genera is plotted over time, for term infants is depicted over age in weeks in the 3 panels, antecubital fossa, forehead and gluteal region respectively from top to bottom. Taxonomic profiling showed that *Flavobacterium* was the dominant genus of the microbiota followed by *Staphylococcus*.

**Figure D: Corrected gestational age and alpha diversity**

**
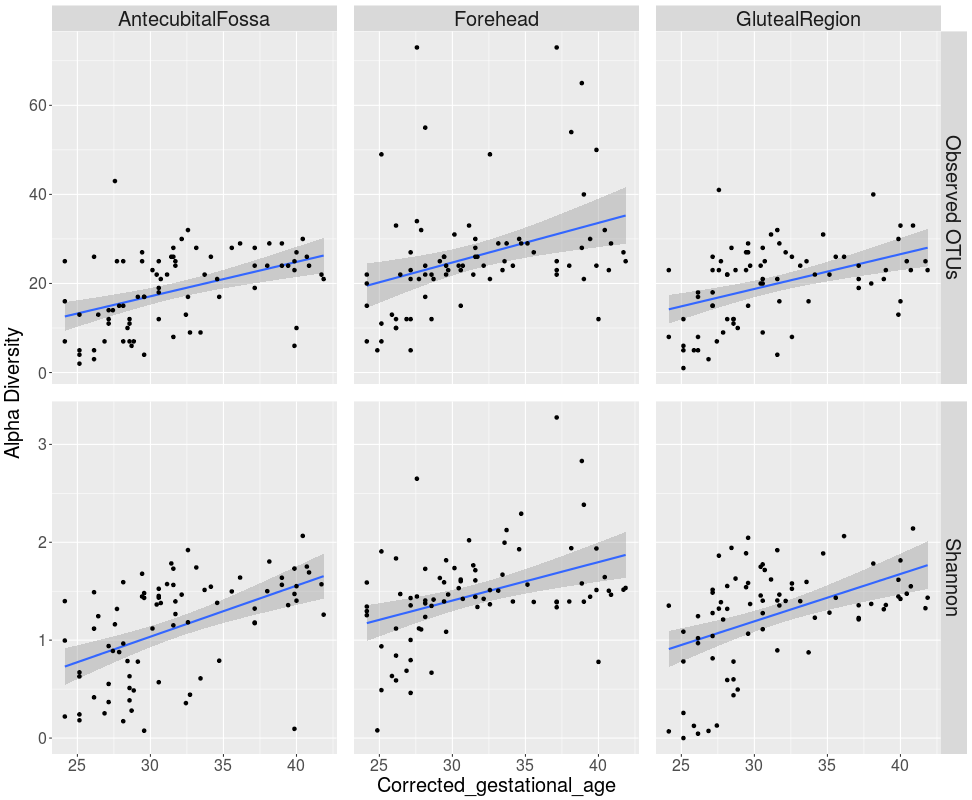
**

**Figure D legend**

**Fig D1** (top panel) Scatter plot of observed OTUs plotted against corrected gestational age (i.e., each sample is assigned a corrected gestational age based on the gestational age at birth + week of sampling. Therefore, an individual contributes multiple data points to each plot) for each of the 3 skin sites. Term infants were sampled at 2 time points (birth and between 2-3 weeks of age) and preterm infants every week from birth for 5 time-points. p-values for the regression coefficient for corrected gestational age in linear mixed effects model for observed OTUs were antecubital fossa (p = 0.001); forehead (p = 0.03); gluteal region (p <0.001). Community abundance in each of the 3 sites is significantly correlated with gestational age.

**Fig D2** (bottom panel) Scatter plot of SDI plotted against corrected gestational age. p-values for the regression coefficient for corrected gestational age in linear mixed effects model for SDI were: antecubital fossa (p<0.001); forehead (p=0.008); gluteal region (p<0.001). Community diversity in each of the 3 sites is significantly correlated with gestational age.

**Figure E: Antibiotic duration and microbial diversity of the cutaneous microbiome**

**Fig E1**


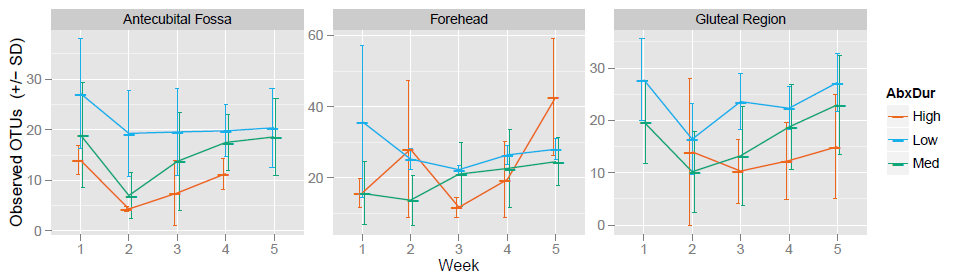


**Fig E2**


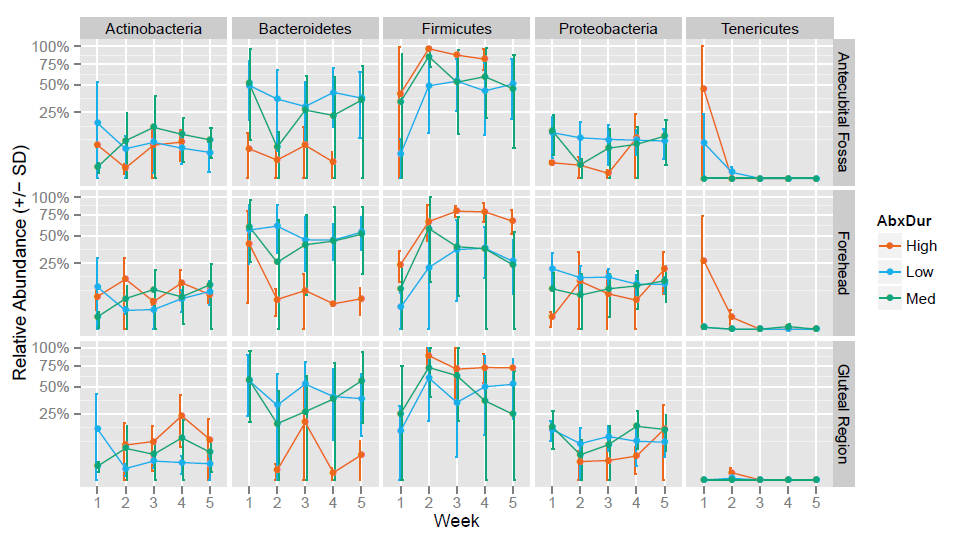


**Figure E legend: Fig E1** shows differences in observed OTUs (mean ± SD) after arbitrary stratification of antibiotic exposure as low (2-10 days), medium (11-14 days) and high (> 14 days). Number of observed OTUs were inversely proportional to the duration of antibiotic therapy.

**Fig E2** Relative abundance of the top 5 most abundant phyla (mean ± SD) is plotted over time after antibiotic exposure stratified as low, medium and high. Taxonomic profiling showed trends of increase in Firmicutes and decrease in Bacteroidetes with increasing duration of antibiotic exposure.

**Figure F- Differences in alpha diversity measures by feeding** **methods in preterm neonates**

**Figure F legend: Fig F** shows the differences in alpha diversity indices of community abundance (OTUs-Figs F1, F3 and F5 of antecubital fossa, forehead and gluteal region respectively) and diversity (SDI- Figs F2, F4 and F6 of antecubital fossa, forehead and gluteal region respectively ), among preterm neonates who were fed predominantly (> 50% of feed intake) with maternal expressed breast milk (MEBM), donor expressed breast milk (DEBM), formula and those administered total parenteral nutrition (TPN). Higher OTUs and SDI was observed in groups that were fed predominantly with formula and MEBM. Data shown in mean and standard error of the mean.

**Figure G: Differences in alpha diversity measures by mode of delivery in preterm neonates**

**Figure G legend: Fig G** shows the differences in alpha diversity indices of community abundance (OTUs- Figs G1, G3 and G5 of antecubital fossa, forehead and gluteal region respectively) and diversity (SDI- Figs G2, G4 and G6 of antecubital fossa, forehead and gluteal region respectively), among preterm neonates who were delivered by vaginal or C-section. Data shown in mean and standard error of the mean. Fig G shows alpha diversity indices of OTUs and Shannon diversity index are not significantly different by mode of delivery (C-section or vaginal) in the neonatal period.

**Table A**. **Characteristics of enrolled patients**

|  | **BW (g)** | **Sex** | **GA**  **(wks)** | **Place** | **Del** | **CAM** | **Initial Abx** | **Abx (days)** | **Nutrition** | **Late-onset**  **Sepsis** | **NEC Stage II or III** |
| --- | --- | --- | --- | --- | --- | --- | --- | --- | --- | --- | --- |
| 1 | 2690 | F | 37 4/7 | NBN | CS | no | no | 0 | Formula | no | no |
| 2 | 1360 | M | 32 1/7 | NICU | CS | no | yes | 2 | Formula | no | no |
| 3 | 3230 | F | 39 | NBN | Vag | no | no | 0 | Breast feeding | no | no |
| 4 | 3605 | M | 39 3/7 | NBN | Vag | no | no | 0 | Breast feeding | no | no |
| 5 | 3668 | F | 40 5/7 | NBN | CS | no | no | 0 | Breast feeding | no | no |
| 6 | 3755 | M | 38 6/7 | NBN | Vag | no | no | 0 | Breast feeding | no | no |
| 7 | 2724 | M | 38 | NBN | Vag | no | no | 0 | Breast feeding | no | no |
| 8 | 3600 | M | 39 6/7 | NBN | Vag | no | no | 0 | Breast feeding | no | no |
| 9 | 860 | F | 29 3/7 | NICU | CS | no | no | 2 | Breast milk (DEBM/EBM),  PN for 13 days | no | no |
| 10 | 820 | M | 27 5/7 | NICU | CS | no | yes | 8 | Breast milk (EBM/DEBM),  PN-14 days | *Enterobacter aerogenes* | no |
| 11 | 890 | F | 26 3/7 | NICU | Vag | no | no | 2 | Breast milk (Donor EBM/EBM) | no | no |
| 12 | 669 | F | 24 1/7 | NICU | CS | yes | yes | 15 | PN and very little feeds | CONS | yes |
| 13 | 751 | M | 24 1/7 | NICU | CS | yes | yes | 9 | PN and very little feeds | CONS | yes |
| 14 | 706 | M | 24 1/7 | NICU | CS | yes | yes | 15 | PN and very little feeds | CONS | yes, perforation |
| 15 | 2994 | F | 39 | NICU | Vag | no | yes | 4 | Breast feeding | no | no |
| 16 | 791 | F | 24 6/7 | NICU | Vag | no | yes | 4 | Donor EBM | MSSA subcutaneous abscess | no |
| 17 | 3180 | F | 38 6/7 | NICU | CS | no | no | 0 | Formula | no | no |
| 18 | 2475 | M | 37 1/7 | NICU | Vag | no | yes | 2 | PN | no | no |
| 19 | 1195 | M | 28 4/7 | NICU | CS | no | yes | 21 | Breast milk, EBM/DEBM PN- 17 days | no | no |
| 20 | 1281 | F | 27 4/7 | NICU | CS | no | yes | 2 | Breast milk, PN -10 days | no | no |
| 21 | 1310 | M | 27 4/7 | NICU | CS | no | yes | 2 | Breast milk, PN -11days | no | no |
| 22 | 1417 | M | 30 4/7 | NICU | Vag | yes | yes | 2 | Breast milk | no | no |
| 23 | 740 | M | 24 1/7 | NICU | Vag | no | yes | 10 | PN | *C. albicans* | yes, perforation |
| 24 | 1001 | M | 27 1/7 | NICU | CS | no | yes | 5 | Breast milk (EBM/DEBM),  PN-16 days | no | no |
| 25 | 2469 | M | 37 1/7 | NICU | CS | no | no | 0 | Breast milk | no | no |
| 26 | 1885 | M | 37 1/7 | NICU | CS | no | yes | 2 | Breast milk, PN -14 days | no | no |
| 27 | 3544 | M | 38 6/7 | NICU | Vag | no | no | 2 | Breast milk | no | no |
| 28 | 3785 | F | 37 1/7 | NICU | CS | no | yes | 1 | Breast milk | no | no |
| 29 | 1365 | F | 30 5/7 | NICU | Vag | no | no | 0 | Breast milk (EBM),  PN-5days | no | no |
| 30 | 2985 | M | 39 1/7 | NICU | CS | no | yes | 2 | Breast milk | no | no |

D

**Table A footnote:** BW- birth weight, GA- gestational age in weeks, NBN- newborn nursery followed by home, NICU- neonatal intensive care unit, Del: mode of delivery, CS- cesarean section, Vag- vaginal delivery, CAM- chorioamnionitis, Abx- antibiotics, Initial antibiotics was for first 48 hrs of life, PN- parenteral nutrition, EBM- expressed breast milk, DEBM- donor expressed breast milk, NEC- necrotizing enterocolitis, CONS-Coagulase negative Staphylococcus, MSSA- Methicillin Sensitive *Staphylococcus aureus*.

**Table B. V3V5** Sequence metrics

| Subject cohort | Number of samples | Total samples after quality trimming and filtering | After rarefaction to 1000 reads/sample | Average sequences/sample |
| --- | --- | --- | --- | --- |
| Preterm neonates (n=15)  Term neonates (n=15) | 309 skin swabs from 30 subjects | 280 samples  1, 221, 507 reads | 235 samples from 15 preterm and 14 term infants | Targeted depth  5000 reads/sample |

**Table B- foot note**: Preterm and term cohort samples were of comparable average sequence length, total sequences per sample and average sequences per sample.
